# Assessment of neutrophil-to-lymphocyte ratio, platelet-to-lymphocyte ratio and platelet count as predictors of long-term outcome after R0 resection for colorectal cancer

**DOI:** 10.1038/s41598-017-01652-0

**Published:** 2017-05-04

**Authors:** Corrado Pedrazzani, Guido Mantovani, Eduardo Fernandes, Fabio Bagante, Gian Luca Salvagno, Niccolò Surci, Tommaso Campagnaro, Andrea Ruzzenente, Elisa Danese, Giuseppe Lippi, Alfredo Guglielmi

**Affiliations:** 1 Division of General and Hepatobiliary Surgery, Department of Surgical Sciences, Dentistry, Gynecology and Pediatrics, University of Verona, Verona, Italy; 2 Division of Minimally Invasive, General and Robotic Surgery, University of Illinois at Chicago, Chicago, USA; 3 Section of Clinical Biochemistry, Department of Neurological, Biomedical and Movement Sciences, University of Verona, Verona, Italy

## Abstract

Neutrophil-to-lymphocyte ratio (NLR), platelet-to-lymphocyte ratio (PLR) and platelet count (PC) were shown to be prognostic in several solid malignancies. We analysed 603 R0 resected patients to assess whether NLR, PLR and PC correlate with other well-known prognostic factors and survival of patients with colorectal cancer (CRC). Receiver operating characteristic (ROC) curve analysis was performed to define cut-off values for high and low ratios of these indices. Univariate and multivariate analysis were used to determine the prognostic value of NLR, PLR and PC for overall and cancer-related survival. The distribution of NLR, PLR and PC in CRC patients was compared with 5270 healthy blood donors. The distribution of NLR, PLR and PC was significantly different between CRC patients and controls (all p < 0.05). A significant but heterogeneous association was found between the main CRC prognostic factors and high values of NLR, PLR and PC. Survival appeared to be worse in patients with high NLR with cancers in AJCC/UICC TNM Stages I-IV; nonetheless its prognostic value was not confirmed for cancer-related survival in multivariate analysis. After stratification of patients according to AJCC/UICC TNM stages, high PC value was significantly correlated with overall and cancer-related survival in TNM stage IV patients.

## Introduction

Despite substantial improvement in early diagnosis, surgical techniques and adjuvant therapies, colorectal cancer (CRC) remains the third most commonly diagnosed cancer and the third leading cause of cancer-related mortality worldwide^1^.

The most appropriate management of CRC entails a deep knowledge of the pivotal role played by molecular factors involved in the pathogenesis of this condition. Such knowledge can also help identifying prognostic biomarkers that can predicting the outcome. The prognostic value of many putative biomarkers has been investigated so far^2–4^.

Amongst these, peripheral blood neutrophil-to lymphocyte ratio (NLR), platelet-to-lymphocyte ratio (PLR), as well as platelet count (PC) have recently emerged as potentially useful tests, since their value may mirror a shift of the immune response in patients with colorectal malignancies.

Inflammation plays a crucial role in the pathogenesis and progression of many types of cancer. Some recent studies demonstrated that systemic inflammatory response correlates with postoperative survival in different cancer patients^5, 6^.

Moreover, systemic inflammatory response to tumours is associated with abnormalities of several blood components, especially neutrophils and lymphocytes. Several hypotheses have been proposed to explain the relationship between cancer and increased values of both PC and plasma fibrinogen^7, 8^. More specifically, platelets release angiogenic and putative tumour growth factors such as platelet factor 4 (PF4), transforming growth factor beta (TGF-β) and platelet-derived growth factor (PDGF), all of which promote cancer progression and endotelial cell growth^9–11^.

The aim of this retrospective study was to evaluate the prognostic value of preoperative neutrophils to lymphocyte ratio (NLR), platelet to lymphocyte ratio (PLR) and PC in patients undergoing potentially curative (R0) resection for colorectal cancer.

## Results

### Distribution of NLR, PLR and PC in study group and controls

**Figure 1:**
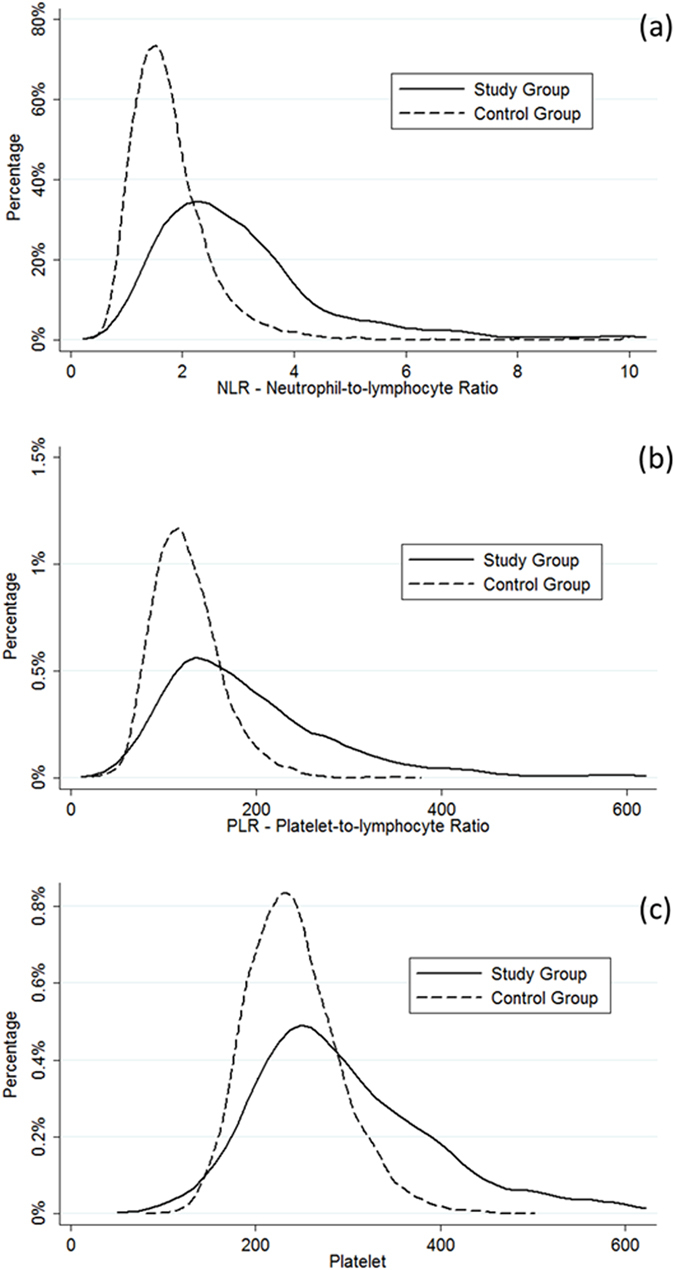
Distribution of (**a**) NLR, (**b**) PLR, and (**c**) PC stratified by study and control groups.

Overall, 603 out of the 1075 patients with CRC observed were finally included in our study according to our inclusion criteria. The distribution of NLR, PLR and PC in cases and controls are shown in Fig. 1. The mean (±SD) preoperative values of the CRC patients were significantly higher than those of the control population (NLR 3.1 ± 1.8 vs. 1.8 ± 1; p < 0.001, PLR 194 ± 98 vs. 126 ± 38; p < 0.001; PC 298 ± 104 × 10^9^/L vs. 241 ± 51 × 10^9^/L; p < 0.001) (Table 1). The following optimal cut-off values were identified: 3.5 for NLR (i.e. low [L]-NLR ≤ 3.5 and high [H]-NLR > 3.5), 350 for PLR (i.e. low [L]-PLR ≤ 350 and high [H]-PLR > 350) and 350 × 10^9^/L for PC (i.e. low [L]-PC ≤ 350 × 10^9^/L and high [H]-PC > 350 × 10^9^/L), respectively (see Supplementary Fig. S1). According to these thresholds, increased values were observed in 26.2% of CRC cases versus 3.2% of the controls for NLR, 6.8% of cases versus 0.0004% of controls for PLR, and 24.9% of cases versus 2.7% of controls for PC (all p < 0.001).

**Table 1:** NLR, PLR and PC of the 603 patients under study and of the 5270 blood donors cases of the control group.

	Patients	Controls	p value
Mean (±SD) age (years)	68.2 (±12.9)	41.2 (±12)	<0.001
Male gender	347 (57.5%)	3946 (74.8%)	<0.001
Mean (±SD) neutrophil count	4.7 (±1.8)	3.6 (±2.9)	<0.001
Mean (±SD) lymphocyte count	1.7 (±0.7)	2 (±0.6)	<0.001
Mean (±SD) PC	298 (±104)	240 (±51)	<0.001
Mean (±SD) NLR	3.1 (±1.8)	1.8 (±1)	<0.001
Mean (±SD) PLR	194 (±98)	126 (±38)	<0.001

### Correlation between NLR, PLR and PC and clinicopathological variables

The correlations of NLR, PLR and PC with clinicopathological variables are shown in Table 2. H-NLR was more frequently observed in patients with increased age (p = 0.026), advanced pT (p < 0.001), TNM stage tumours (p < 0.001), metastatic disease (p < 0.001) and CEA positive cancers (p = 0.017). H-PLR was observed more frequently in advanced pT stage tumours (p = 0.023), although the percentage of patients with H-PLR did not exceeded 12%. H-PC was observed more frequently in the female gender (p = 0.013), colon tumour location (p = 0.006), advanced pT (p < 0.001), TNM stage tumours (p = 0.008) and CEA positive cancer (p = 0.005).

**Table 2:** Correlations between NLR, PLR and PC and main clinicopathological variables for the 603 patients under study.

	No. of patients	H-NLR	H-PLR	H-PC
Age		p = 0.026	p = 0.747	p = 0.398
≤68.9 years	299	66 (22.1%)	19 (6.4%)	79 (26.4%)
>68.9 years	304	92 (30.3%)	22 (7.2%)	71 (23.4%)
Gender		p = 0.263	p = 0.417	p = 0.013
Male	347	97 (28%)	21 (6.1%)	73 (21%)
Female	256	61 (23.8%)	20 (7.8%)	77 (30.1%)
Tumour location		p = 1.000	p = 0.280	p = 0.006
Colon	438	115 (26.3%)	33 (7.5%)	122 (27.9%)
Rectum	165	43 (26.1%)	8 (4.8%)	28 (17%)
Depth of invasion (pT)		p < 0.001	p = 0.023	p < 0.001
pT1	77	7 (9.1%)	0%	8 (10.4%)
pT2	76	22 (28.9%)	5 (6.6%)	12 (15.8%)
pT3	270	63 (23.3%)	16 (5.9%)	70 (25.9%)
pT4a	122	42 (34.4%)	13 (10.7%)	37 (30.3%)
pT4b	58	24 (41.4%)	7 (12.1%)	23 (39.7%)
Node involvement (pN)		p = 0.679	p = 0.374	p = 0.172
pN0	380	95 (25%)	26 (6.9%)	88 (23.2%)
pN1	155	44 (28.4%)	8 (5.2%)	39 (25.2%)
pN2	68	19 (27.9%)	7 (10.3%)	23 (33.8%)
Systemic metastasis (M)		p < 0.001	p = 0.059	p = 0.101
M0	563	137 (24.3%)	36 (6.4%)	136 (24.2%)
M1a	33	15 (45.5%)	3 (9.1%)	10 (30.3%)
M1b	7	6 (85.7%)	2 (28.1%)	4 (57.1%)
AJCC/UICC TNM Stage		p < 0.001	p = 0.106	p = 0.008
Stage I	130	27 (20.8%)	5 (3.8%)	18 (13.8%)
Stage II	237	64 (27%)	21 (8.9%)	65 (27.4%)
Stage III	196	46 (23.5%)	10 (5.1%)	53 (27%)
Stage IV	40	21 (52.5%)	5 (12.5%)	14 (35%)
CEA serum level^b^		p = 0.017	p = 0.818	p = 0.005
≤5 ng/mL	303	75 (24.8%)	21 (6.9%)	69 (22.8%)
>5 ng/mL	92	35 (38%)	7 (7.6%)	35 (38%)

No differences in NLR and PLR were observed according to tumour grading, lymphatic, vascular and perineural invasion. Conversely, H-PC was found to be significantly associated with poor cancer differentiation (G3 tumours: 23.3% vs. 8.8%; p < 0.001), vascular invasion (VI + tumours: 32.2% vs. 20.7%; p = 0.013), perineural invasion (NI + tumours: 32.5% vs. 23.3%; p = 0.041) and mucinous histotype (mucinous tumours: 34.5% vs. 22.9%; p = 0.046). No difference in PC values was observed in relation to lymphatic invasion (p = 0.834) and inflammatory reaction (p = 0.986).

### NLR, PLR and PC and survival analysis

Overall and cancer-related survival rates in relationship to the main clinicopathological variables, NLR, PLR and PC are shown in Table 3.

**Table 3:** Five-year overall and cancer-related survival rates according to the main clinicopathological characteristics, NLR, PLR and PC for the 603 patients under study.

	No. of patients	5-year survival rate	
		Overall	Cancer-related
Age		p < 0.001	p = 0.022
≤68.9 years	299	85.9	89.4
>68.9 years	304	64.6	79.3
Gender		p = 0.055	p = 0.052
Male	347	73.7	82.6
Female	256	77.2	87.7
Tumour location		p = 0.699	p = 0.818
Colon	438	74.8	84.1
Rectum	165	76.1	86.4
Depth of invasion (pT)		p < 0.001	p < 0.001
pT1	77	89.9	100
pT2	76	86.5	90.9
pT3	270	80.8	90.8
pT4a	122	54.5	65.2
pT4b	58	55.2	61.5
Node involvement (pN)		p < 0.001	p < 0.001
pN0	380	81.6	92.5
pN1	155	71.1	78.6
pN2	68	44.7	46.9
Systemic metastasis (M)		p < 0.001	p < 0.001
M0	563	77.8	87.8
M1a	33	46.5	50.8
M1b	7	0	0
AJCC/UICC TNM Stage		p < 0.001	p < 0.001
Stage I	130	89.2	97
Stage II	237	79.1	91.6
Stage III	196	68	75.5
Stage IV	40	37.9	45.8
CEA serum level^a^		p < 0.001	p < 0.001
≤5 ng/mL	303	78.6	86.1
>5 ng/mL	92	58.3	73.2
Neutrophil-to-lymphocyte ratio		p < 0.001	p = 0.022
L-NLR	445	79.8	86.6
H-NLR	158	62.5	79.4
Platelet-to-lymphocyte ratio		p = 0.070	p = 0.239
L-PLR	562	75.9	86.6
H-PLR	41	65.9	79.4
Platelet count		p = 0.122	p = 0.151
L-PC	453	76.3	85.2
H-PC	150	71.8	83.5

^a^CEA serum level was available for 401 patients.

**Figure 2:**
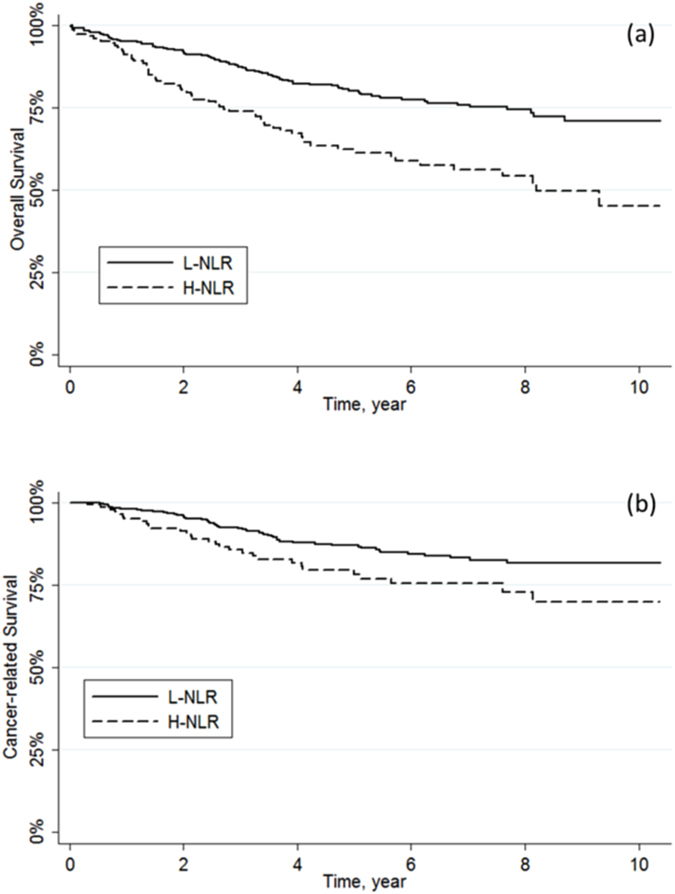
Kaplan-Meier curves for (**a**) overall survival (p < 0.001) and (**b**) cancer-related survival (p = 0.022) stratified by NLR.

Age, depth of tumour invasion (pT), node involvement (pN), metastatic disease (M), TNM stage and CEA positivity were confirmed to be significant predictors of overall and cancer-related survival. Patients with H-NLR had lower 5-year overall survival (p < 0.001) and worse cancer-related survival rate (p = 0.020) (Fig. 2). No differences in overall and cancer-related survival were observed between high and low values of both PLR and PC (Table 3) (see Supplementary Figs S2 and S3).

In the Cox regression model, H-NLR, H-PLR and H-PC were found to be independent predictors of 5-year overall survival but not cancer-related survival after multiple adjustments (Table 4) (see Supplementary Tables S1 and S2).

**Table 4:** Multivariable survival analysis including neutrophil-to-lymphocyte ratio (NLR) for the 603 patients under study.

	Overall Survival		Cancer-related Survival	
	HR (95% CI)	p value	HR (95% CI)	p value
Age (years)		<0.001		0.017
≤68.9 years	—		—	
>68.9 years	2.98 (2.07–4.29)		1.69 (1.09–2.61)	
Gender		0.10		0.08
Female	—		—	
Male	1.33 (0.95–1.87)		1.50 (0.96–2.36)	
Histological Type		0.46		0.10
Adenocarcinoma	—		—	
Mucinous	0.85 (0.56–1.30)		0.61 (0.33–1.01)	
TNM Stage		<0.001		<0.001
Stage I	—		—	
Stage II	1.59 (0.90–2.82)		2.73 (0.92–8.13)	
Stage III	2.92 (1.66–5.13)		9.65 (3.43–27.14)	
Stage IV	6.49 (3.45–12.21)		29.78 (10.22–86.75)	
Neutrophil-to-lymphocyte ratio		0.003		0.40
L-NLR	—		—	
H-NLR	1.15 (0.86–1.54)		1.22 (0.77–1.93)	

^a^Values in parentheses are 95% confidence intervals. Hazard ratio and p values were derived from Cox regression analysis, controlling for all other variables.

**Figure 3:**
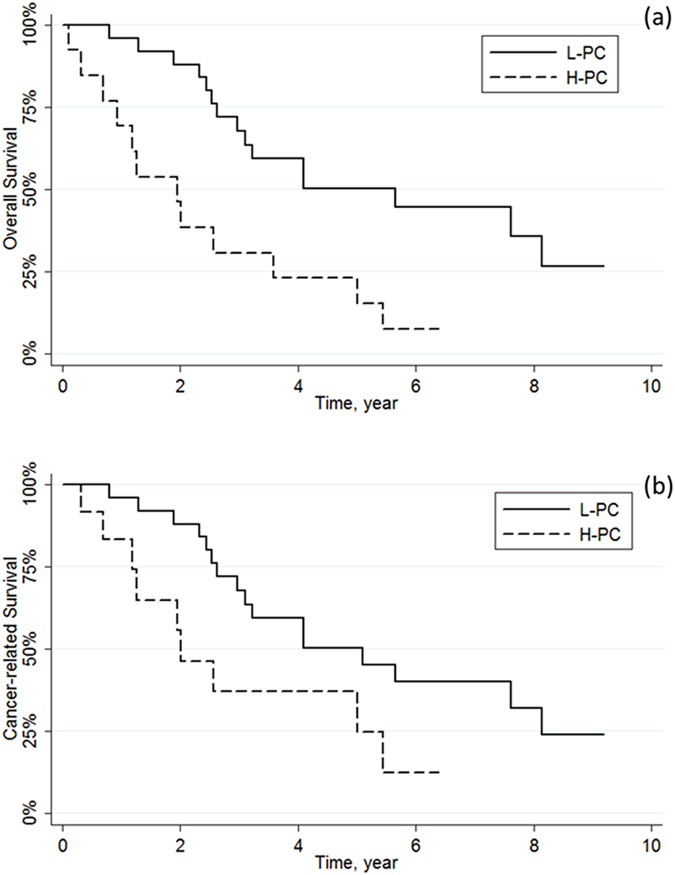
Kaplan-Meier curves for (**a**) overall survival (p = 0.002) and (**b**) cancer-related survival (p = 0.041) stratified by PC in AJCC/UICC TNM Stage IV.

To further investigate the prognostic role of NLR, PLR and PC, the survival rates were stratified according to AJCC/UICC TNM stages. No differences in 5-year overall and cancer-related survival were observed between high and low values of both NLR and PLR across TNM stages I to IV (all p > 0.05). Similarly, no differences in 5-year overall and cancer-related survival were observed between high and low values of PC among TNM stages I to III (all p > 0.05). Notably, H-PC was found to be a negative predictor of overall and cancer-related survival compared to L-PC in patients with AJCC/UICC TNM stage IV tumours (overall survival, p = 0.002; cancer-related survival, p = 0.041) (Fig. 3).

## Discussion

Increased values of both NLR and PLR were recently found to be negative predictors of survival in several malignancies such as oesophageal, hepato-bilio-pancreatic prostate cancer^12–16^ as well as CRC^6, 17, 18^.

The main findings of this study are: (1) the distribution of NLR, PLR and PC was found to be significantly different between CRC patients and controls; (2) a significant but variable association was found between the main known CRC prognostic factors and H-NLR, H-PLR and H-PC; (3) H-NLR was associated with significantly poorer overall survival in univariate as well multivariate analysis, but it had no impact on cancer-related survival; (4) after stratification of patients according to AJCC/UICC TNM stage, H-PC was significantly related to overall and cancer-related survival.

Although the clinical significance of NLR is still unclear, it has been pointed out that this parameter may globally reflect a shift of the immune response towards a pro-inflammatory pattern (i.e. high value of neutrophils) balanced by a depression of cell-mediated immunity (i.e. low value of lymphocytes). Since platelets are active players in inflammatory response, both thrombocytosis and high PLR are probably part of the same pathophysiology process^13^. Cancer cells produce many pro-inflammatory cytokines and chemokines such as GCSF, IL-1 and IL-6, but they are also actively involved in cytotoxic T-cell and natural killer cell suppression^19^. Altogether these aspects may contribute to generate a pro-inflammatory and immunosuppressive milieu^20^.

Interestingly, Turner *et al*. recently analysed local chronic inflammatory cell infiltrate together with systemic inflammation (i.e. NLR) in stage II CRC and showed that the combination of the two parameters have an increased prognostic value, which is also independent from standard clinical and pathological criteria. In this experience, cases with high intratumoural inflammation and low systemic inflammation showed a significantly better prognosis, whilst cases with low intratumoural inflammation and high systemic inflammatory response had a worse one^18^.

Beside cancer cells, solid tumours are composed of heterogeneous cell populations, including endothelial cells, fibroblasts and pericytes. Different cells produce several mediators capable of recruiting different leukocytes populations from the circulation to the tumour site. Within solid tumours, the myeloid population is certainly one of the most represented. Amongst these cells, tumour associated monocytes (TAM) and immature myeloid-derived suppressor cells (MDSC) play several important functions such as immunosuppression, angiogenesis tumour growth and cancer dissemination^21, 22^. It is possible that larger, less differentiated and more advanced tumours, (T stage but also N and M stages), bear more effective chemotactic activity towards the myeloid lineage, which in turn promotes progression of the cancer. It is reasonable to suggest that each individual immune response to cancer cells may contribute to define part of the biological behaviour of a malignancy. In this respect, it is also conceivable that a lower intratumoural inflammation and a higher systemic inflammatory response might translate in a decreased immunological local control, so engendering a systemic pro-inflammatory environment which ultimately facilitates cancer progression. Factors that could drive one or another type of immune response remain unknown so far.

The significant difference of NLR, PLR and PC values between CRC patients and healthy controls is one of the more interesting findings of our study. The sample size of the control population in our investigation is the largest in which NLR and PLR have ever been assessed, and this contributes to reinforce the clinical significance of our data.

Although the mean patient age was different between control and study groups, NLR, PLR and PC did not change in parallel to the increased age of the patients (Supplementary Figs S4 and S5). The higher frequency of H-NLR in patients with an age over the median value is likely due to the higher number of advanced pT and TNM stage tumours in this specific group (P < 0.005, data not shown).

Notably, the cut-off values of NLR, PLR and PC that we have calculated (i.e. 3.5, 350 and 350 × 10^9^/L) partially differ from those reported in other investigations. This is not surprising since this difference may be due to the analysis of a different study population or to the use of a different haematological analyser, highlighting the importance of local calculation of predictive thresholds for these parameters.

In our study, H-NLR, H-PLR and H-PC were found to be significantly associated with some well-known CRC prognostic factors. More specifically, H-NLR correlated with pT, TNM stage, presence of metastatic disease and CEA positivity. H-PLR was associated with advanced pT tumours, whereas H-PC correlated with female gender and colon tumour location. A similar correlation between NLR and tumour burden was previously described. More specifically, in a retrospective study including 504 patients with stage II and III colon cancer, Absenger *et al*. found that NLR independently predicted the time to recurrence^23^.

Another study^24^ described a significant correlation between H-NLR, size (pT) and metastatic disease burden, although the cut-off value was slightly lower than that identified in our investigation (i.e. 3.0 versus 3.5). NLR was also found to be an independent predictor of worse outcome within the same stage cancer (stage IIA), proving to be an important factor in the evaluation of adjuvant therapy^25^.

H-PC was an independent predictor factor of both overall and cancer-related survival. Interestingly, H-PC was found to be an independent predictor factor of both 5-year overall and cancer-related survival only in patients with stage IV tumours.

The prognostic value of PC in CRC has been previously investigated. Although some results were generated in retrospective or underpowered studies, thrombocytosis at the time of diagnosis of CRC has been convincingly associated with worse disease free and overall survival^8^. Renal^26^, gynaecological^27^ and lung cancers^28, 29^ have also shown to have an elevated PC at the time of diagnosis. Finally, thrombocytosis could identify a patient subgroup in whom cytokine activation is driving a more aggressive disease course, but which is sensitive to therapeutic intervention. Subgroup analyses of the COIN trial on metastatic CRC suggested that patients with normal baseline platelet counts could gain the benefits of intermittent chemotherapy without detriment in survival, whereas those with raised baseline platelet counts have impaired survival and quality of life with intermittent chemotherapy and should not receive a treatment break^30^.

Regarding lymph node involvement, we failed to find a significant correlation with NLR, PLR and PC. This finding is not in line with previous studies, wherein Chiang *et al*. reported that patients with advanced pN stage also had significantly higher NLR values^31^. This difference cannot be easily explained, especially considering that the lymph node yield in our study was adequate, with a mean number of 21.4 harvested lymph nodes.

A potential explanation for the observed correlation between predictors of CRC and NLR, PLR or PC can be brought back to the putative immunological activities of cancer cells.

In particular, the correlation between H-NLR and the presence of systemic metastases can be explained by the fact that a high neutrophil count was found in our CRC patients, and these blood cells actively release cytokines and circulating vascular endothelial growth factor (VEGF), two molecules deeply involved in angiogenesis, cancer growth and metastasis^32^. On the other hand, lymphocytes play a pivotal role in tumour suppression, by inducing cytotoxic cell death and cytokine production, which may ultimately contribute to inhibiting the proliferation and metastatic activity of cancer cells^33, 34^.

The biological background of the observed correlation between lymph node involvement and NLR, PLR or PC remains unexplained. Although Pine *et al*. previously found a correlation between high NLR and pN stage, this study also included non-curative resection and less than 15 lymph nodes were detected in half of the patients^3^.

Therefore, we tend to believe that the prognostic significance of these tests may be stronger for certain characteristics of cancer progression such as depth invasion and systemic metastasis rather than for node involvement.

When we considered Stage I-IV tumours, survival appeared to be worse in patients with H-NLR. This intriguing finding may be partly explained by the differences observed in cancer volume between the L-NLR and H-NLR groups, despite the fact that the prognostic significance of NLR for cancer-related survival could not be confirmed in multivariate analysis.

Unlike NLR and PLR, PC was significantly associated with 5-year overall and cancer-related survival after stratification of patients according to TNM stage. This is also reasonable since platelets may contribute to cancer development by promoting neoangiogenesis, increasing both microvessel permeability and extravasation of cancer cells, producing growth factors (e.g. VEGF, PDGF and TGF-β) and facilitating the interaction between cancer cells and endothelia at metastatic sites^35, 36^.

In conclusion, our study clearly demonstrates that the distribution of NLR, PLR and PC significantly differs between CRC patients and healthy subjects. This difference is more marked in advanced tumours, although it does not unequivocally reflect cancer-related survival. Further studies will be needed to establish the real cost-effectiveness of routinely using these inexpensive, readily available and reproducible biomarkers for establishing the prognosis after R0 resection for CRC.

## Methods

### Patients and eligibility criteria

The original patient population consisted of all patients undergoing surgery for CRC at the Division of General Surgery A, University of Verona Hospital Trust, between January 2005 and December 2013. Inclusion criteria were: elective surgery for histology-proven CRC and age of 18 years or older.

Patients with evidence of infections or other inflammatory conditions during preoperative evaluation were excluded, as well as those who underwent preoperative chemo or radiotherapy, emergency surgery and an R1 or R2 resection. All methods used in this study were performed in accordance with the relevant ethical guidelines and regulations of the University Hospital of Verona, where the investigation was carried out. Informed consent was obtained from all patients and the study protocol was approved by the local ethical committee (ID number: 42763-CRINF-1034 CESC).

### Preoperative work-up and histopathological staging

Prior to surgery, all patients were staged with colonoscopy, computed tomography (CT) of chest-abdomen and pelvis and measurement of cancer biomarkers including carcinoembryonic antigen (CEA) and cancer antigen 19-9 (CA 19-9). Additional imaging studies including magnetic resonance imaging (MRI) and endoluminal ultrasonography were used for staging rectal cancer. Liver MRI and positron emission tomography were also used to evaluate dubious lesions.

Pathology specimens were analysed in accord with the 7^th^ Edition of the American Joint Committee on Cancer (AJCC) and the Union International Contre Le Cancer (UICC) criteria.

### Preoperative assessment of laboratory data

Neutrophil count, lymphocyte count and PC were obtained in venous blood within 2 weeks from the date of surgery. NLR and PLR were calculated by dividing the absolute number of neutrophils or platelets by the absolute number of lymphocytes, respectively.

Blood samples were drawn by an expert phlebotomist in vacuum blood tubes containing K_2_-EDTA (Terumo Europe NV, Leuven, Belgium). The complete blood cell count (CBC) was performed using Advia 2120 (Siemens Healthcare Diagnostics, Tarrytown NY, USA). The local reference ranges are 150–400 × 10^9^/L for platelets, 4.3–10.0 × 10^9^/L for total white blood cells (WBC), 2.0–7.0 × 10^9^/L for neutrophils and 0.95–4.5 × 10^9^/L for lymphocytes, respectively. The same analyser was used throughout the study period. The quality and comparability of test results was validated by data of both internal quality control (IQC) and external quality assessment (EQA).

### Extent of surgery

The complete excision of the tumour burden (R0 resection) was the main outcome of surgery. The extent of surgery was planned according to patient conditions, tumour location and stage. Standard CRC resections (i.e. right hemicolectomy, extended right hemicolectomy, left hemicolectomy, anterior resection, low anterior resection, abdominoperineal resection) with ligation of vessels at their origin were usually performed in order to harvest an adequate number of lymph nodes^37^. The mean number of lymph nodes analyses was 21.4 (SD, 11.4), with only 13.8% of cases having less than 12 lymph nodes excised. In patients affected by synchronous liver metastases or peritoneal disease, liver resection or partial peritonectomy was needed to achieve R0 resection.

### Follow-up and statistical analysis

All clinical and pathological data were retrospectively collected and stored in a digital database. Demographic, clinical, surgical and pathology variables were analysed.

On preliminary analysis, preoperative NLR, PLR and PC were found to be normally distributed. The optimal cut-off values for NLR, PLR and PC as dichotomous predictors of survival was chosen after considering (i) conventional receiver operating characteristics (ROC) curve analysis using death as the outcome (see Supplementary Fig. S1); (ii) Kaplan-Meier curves and proportional hazards regression with cut-off increased in following steps and results recalculated at each step in order to identifying the threshold associated with the greatest separation of curves with the lowest p value; (iii) evaluation of cut-offs proposed by previous literature. The distribution of NLR, PLR and PC in the final study population was also compared with the distribution of these parameters in a total number of 5270 healthy blood donors who had their blood collected during the same period (i.e. control group). The difference was analysed with ANOVA test (Table 1 and Fig. 1); the relationship between study variables (i.e. NLR, PLR and PC) and age was tested using linear regression analysis (see Supplementary Figs S4 and S5).

The significance of differences was evaluated with chi square test or Fisher’s exact test for categorical data (using NLR, PLR and PC as dichotomous variables), and Student’s t-test for continuous variables.

The association of NLR, PLR and PC with overall survival and cancer-related survival was analysed using Kaplan-Meier curves and compared with the log-rank test. The time of survival was measured between the date of surgery and the date of the most recent follow-up examination or death. Multivariable analysis for overall survival and cancer-related survival was performed using Cox regression model by separately considering NLR, PLR and PC values above or below the relative cut-offs and adjusting for the following risk factors: age (>median vs. ≤median), gender (male vs. female), tumour location (rectum vs. colon), histological type (mucinous vs. non-mucinous type) and AJCC/UICC TNM stage (stage II, stage III and stage IV vs. stage I). A p value < 0.05 was considered to be statistically significant.

Statistical analysis was performed using SPSS software version 21.0 version (IBM Corporation, Armonk, NY) and STATA software (Stata Corporation, 2011, MP-Parallel Edition).

## Electronic supplementary material

Supplementary material
