# Supplementary material for: Assessment of neutrophil-to-lymphocyte ratio, platelet-to-lymphocyte ratio and platelet count as predictors of long-term outcome after R0 resection for colorectal cancer
Source: Sci Rep. 2017 May 4;7:1494. doi: 10.1038/s41598-017-01652-0 (PMC5431463; doi:10.1038/s41598-017-01652-0)
Supplement: Supplementary file 1 — Supplementary material [file 41598_2017_1652_MOESM1_ESM.pdf]

Title: Assessment of neutrophil-to-lymphocyte ratio, platelet-to-lymphocyte ratio and platelet count as predictors of long-term outcome after R0 resection for colorectal cancer.

Authors: Corrado Pedrazzani,*[1] Guido Mantovani,[1] Eduardo Fernandes,[2] Fabio Bagante,[1] Gian Luca Salvagno,[3] Niccolò Surci,[1] Tommaso Campagnaro,[1] Andrea Ruzzenente,[1] Elisa Danese,[3] Giuseppe Lippi,[3] Alfredo Guglielmi.[1]

Authors' affiliation: [1]Division of General and Hepatobiliary Surgery, Department of Surgical Sciences, Dentistry, Gynecology and Pediatrics, University of Verona, Verona, Italy; [2]Division of Minimally Invasive, General and Robotic Surgery, University of Illinois at Chicago, Chicago, US; [3]Section of Clinical Biochemistry, Department of Neurological, Biomedical and Movement Sciences, University of Verona, Verona, Italy

Correspondence to: Dott. Corrado Pedrazzani, Chirurgia Generale e Epatobiliare, Policlinico "G.B. Rossi", Piazzale Scuro 10, 37134 Verona, Italia. Tel: ++39 (0)45 8124464-4655; Fax: ++39 (0)45 8027426; E-mail: corrado.pedrazzani@univr.it

Table S1. Multivariable survival analysis including platelet-to-lymphocyte ratio (PLR) for the 603 patients under study.

		Overall Survival		Cancer-related Survival	
		HR (95% CI)	p value	HR (95% CI)	p value
Age (years)			<0.001		0.013
	≤ 68.9 years	-		-	
	> 68.9 years	3.14 (2.18-4.53)		1.75 (1.13-2.71)	
Gender			0.058		0.058
	Female	-		-	
	Male	1.39 (0.99-1.95)		1.56 (0.99-2.45)	
Histological Type			0.66		0.11
	Adenocarcinoma	-		-	
	Mucinous	0.91 (0.59-1.38)		0.62 (0.34-1.12)	
TNM Stage			<0.001		<0.001
	Stage I	-		-	
	Stage II	1.55 (0.87-2.74)		2.67 (0.89-7.96)	
	Stage III	2.83 (1.61-4.97)		9.59 (3.41-26.99)	
	Stage IV	7.11 (3.79-13.33)		30.63 (10.58-86.67)	
Platelet-to-lymphocyte ratio			0.034		0.22
	L-PLR	-		-	
	H-PLR	1.86 (1.05-3.32)		1.64 (0.74-3.62)	

.

[a] Values in parentheses are 95% confidence intervals. Hazard ratio and p values were derived from Cox regression analysis, controlling for all other variables.

Table S2. Multivariable survival analysis including platelet count (PC) for the 603 patients under study.

		Overall Survival		Cancer-related Survival	
		HR (95% CI)	p value	HR (95% CI)	p value
Age (years)			<0.001		0.013
	≤ median	-		-	
	> median	3.15 (2.18-4.53)		1.73 (1.12-2.67)	
Gender			0.033		0.046
	Female	-		-	
	Male	1.45 (1.03-2.05)		1.59 (1.01-2.51)	
Histological Type			0.49		0.09
	Adenocarcinoma	-		-	
	Mucinous	0.86 (0.57-1.31)		0.59 (0.33-1.08)	
TNM Stage			<0.001		<0.001
	Stage I	-		-	
	Stage II	1.53 (0.86-2.71)		2.59 (0.87-7.71)	
	Stage III	2.76 (1.57-4.85)		9.18 (3.26-25.84)	
	Stage IV	6.81 (3.62-12.8)		30.06 (10.37-87.14)	
Platelet count			0.036		0.07
	L-PC	-		-	
	H-PC	1.48 (1.03-2.13)		1.53 (0.96-2.45)	

.

[a] Values in parentheses are 95% confidence intervals. Hazard ratio and p values were derived from Cox regression analysis, controlling for all other variables.

Figure legends

Figure S1. Receiver Operating Characteristic curves plotting the true-positive rate (sensitivity) against the false-positive rate (1-specificity) at various threshold of a) NLR, b) PLR, and c) PC as predictors of overall survival.

Figure S2. Kaplan-Meier curves for a) overall survival ($p=0.070$) and b) cancer-related survival ($p=0.239$) stratified by PLR

Figure S3. Kaplan-Meier curves for a) overall survival ($p=0.122$) and b) cancer-related survival ($p=0.151$) stratified by PC

Figure S4. Linear regression analysis modelling the relationship between age and NLR in a) CRC patients ($r=0.076$; $r^2=0.006$; $p=0.063$) and b) control population ($r=0.008$; $r^2=0.000$; $p=0.560$)

Figure S5. Linear regression analysis modelling the relationship between age and PLR in a) CRC patients ($r=0.015$; $r^2=0.000$; $p=0.727$) and b) control population ($r=0.068$; $r^2=0.005$; $p<0.001$)

Figure S6. Linear regression analysis modelling the relationship between age and PC in a) CRC patients ($r=0.155$; $r^2=0.024$; $p<0.001$) and b) control population ($r=0.056$; $r^2=0.003$; $p<0.001$)

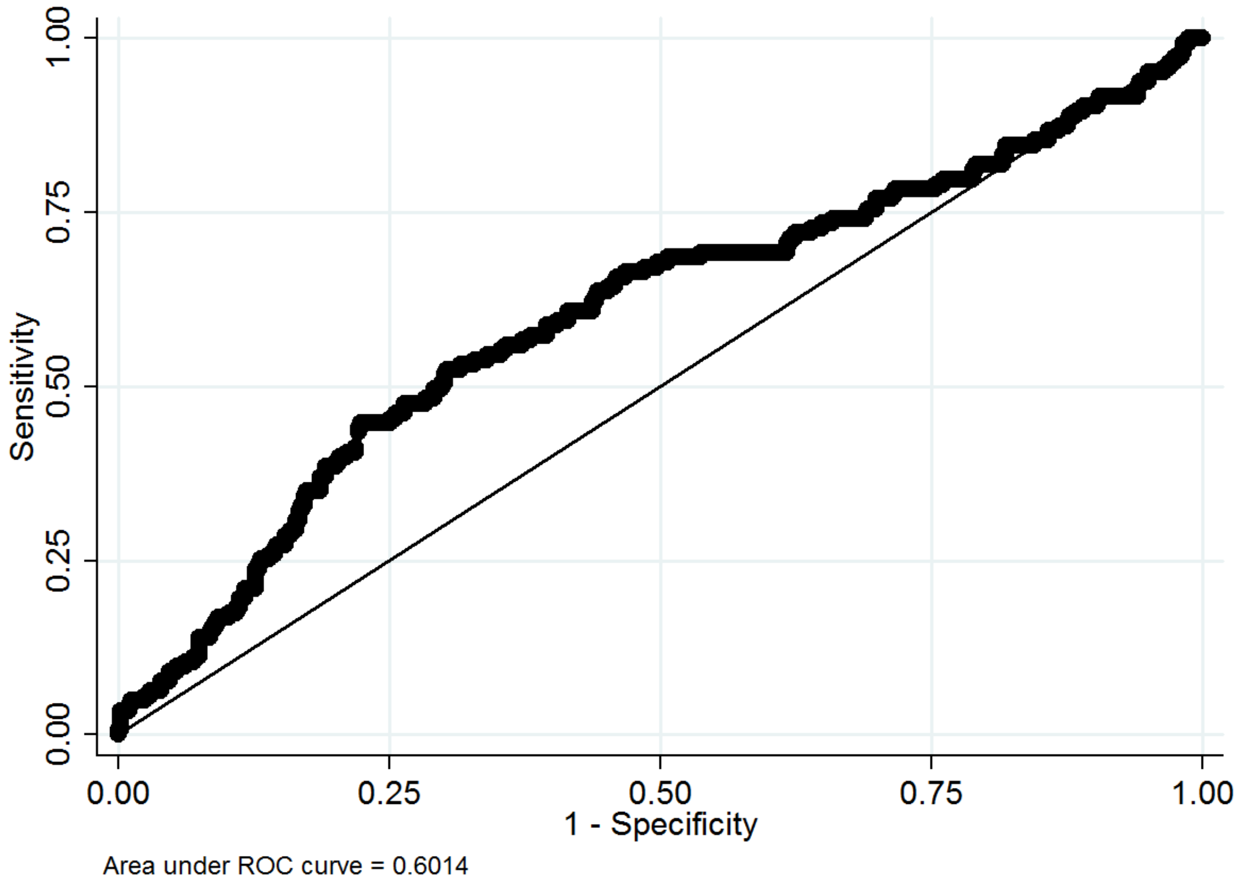
Sensitivity
0.00
0.25
0.50
0.75
1.00
1 - Specificity
Area under ROC curve = 0.6014

Figure S1(a)

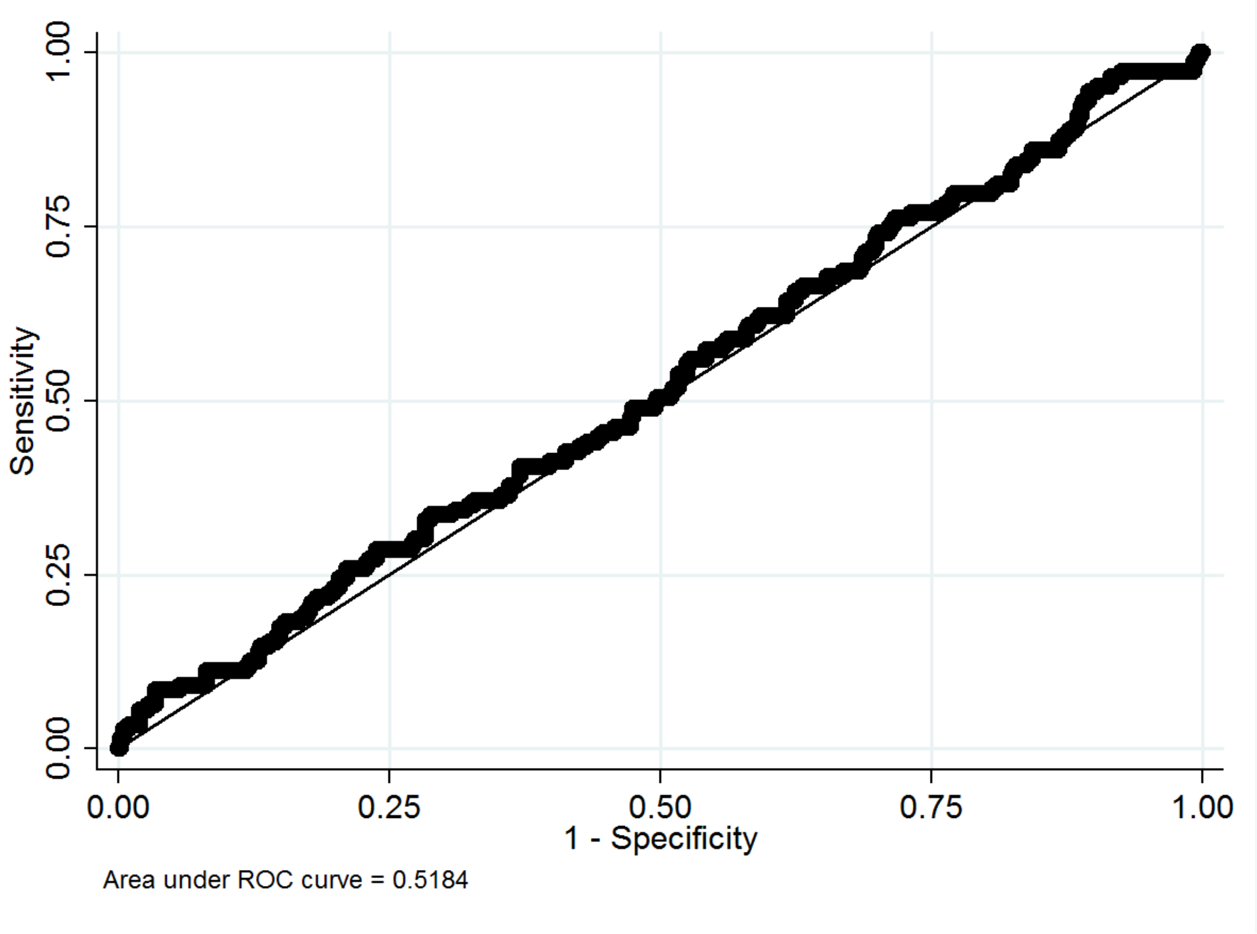
1.00
0.75
0.50
0.25
0.00
Sensitivity
0.00
0.25
0.50
0.75
1.00
1 - Specificity
Area under ROC curve = 0.5184

Figure S1(b)

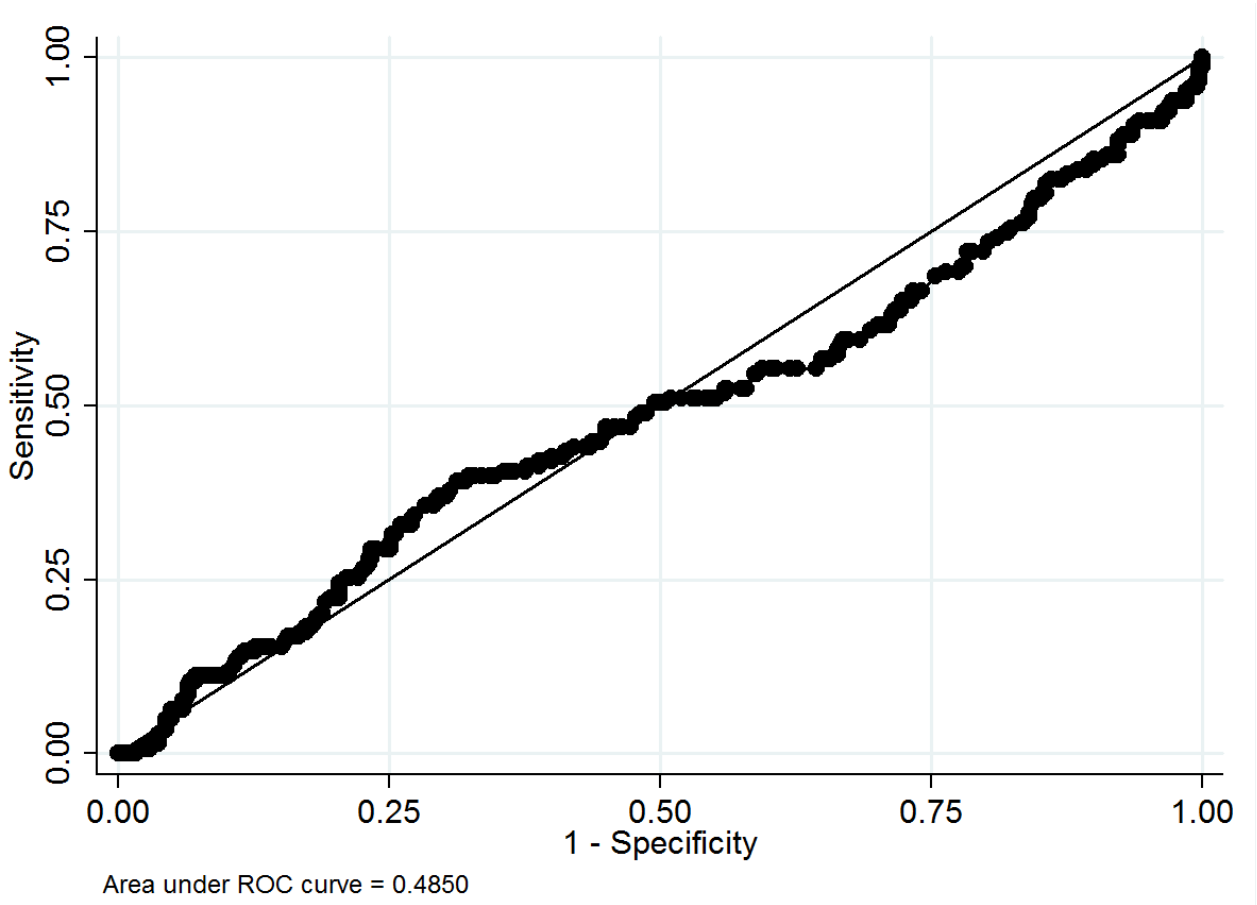
Sensitivity
1 - Specificity
0.00
0.25
0.50
0.75
1.00
Area under ROC curve = 0.4850

Figure S1(c)

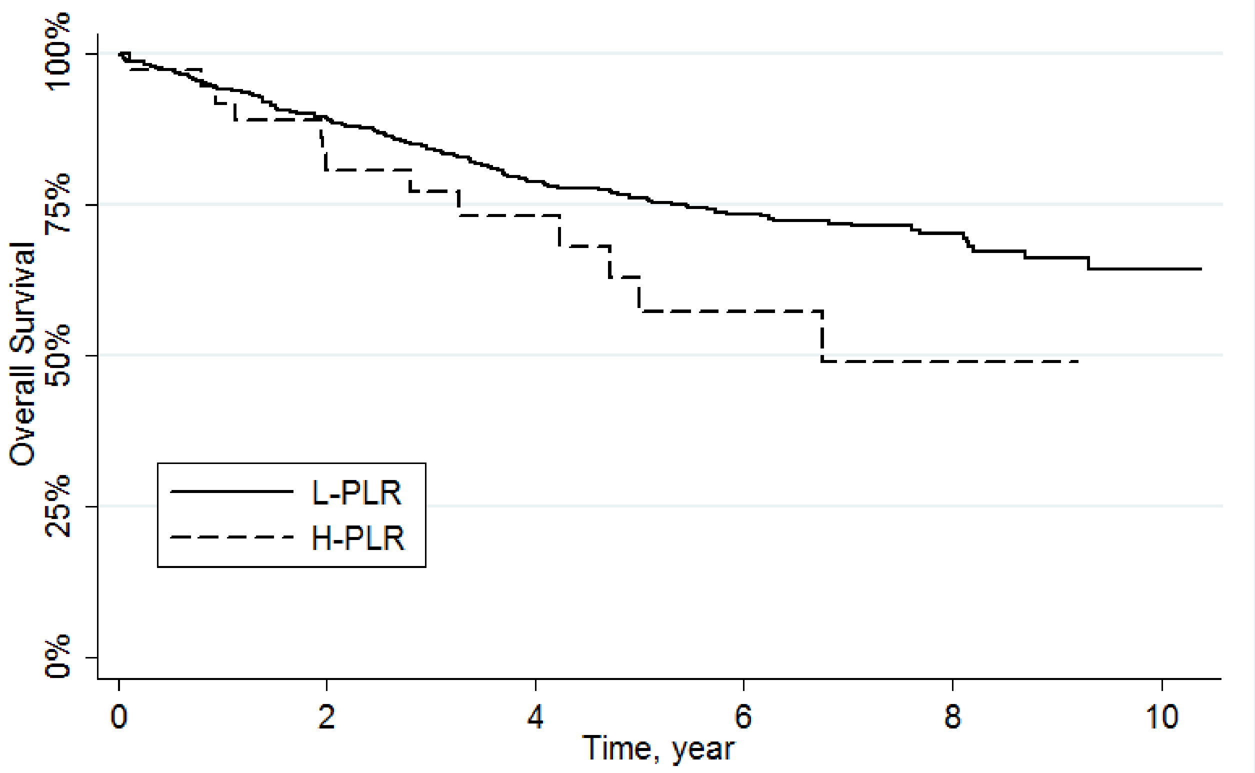

Figure S2(a)

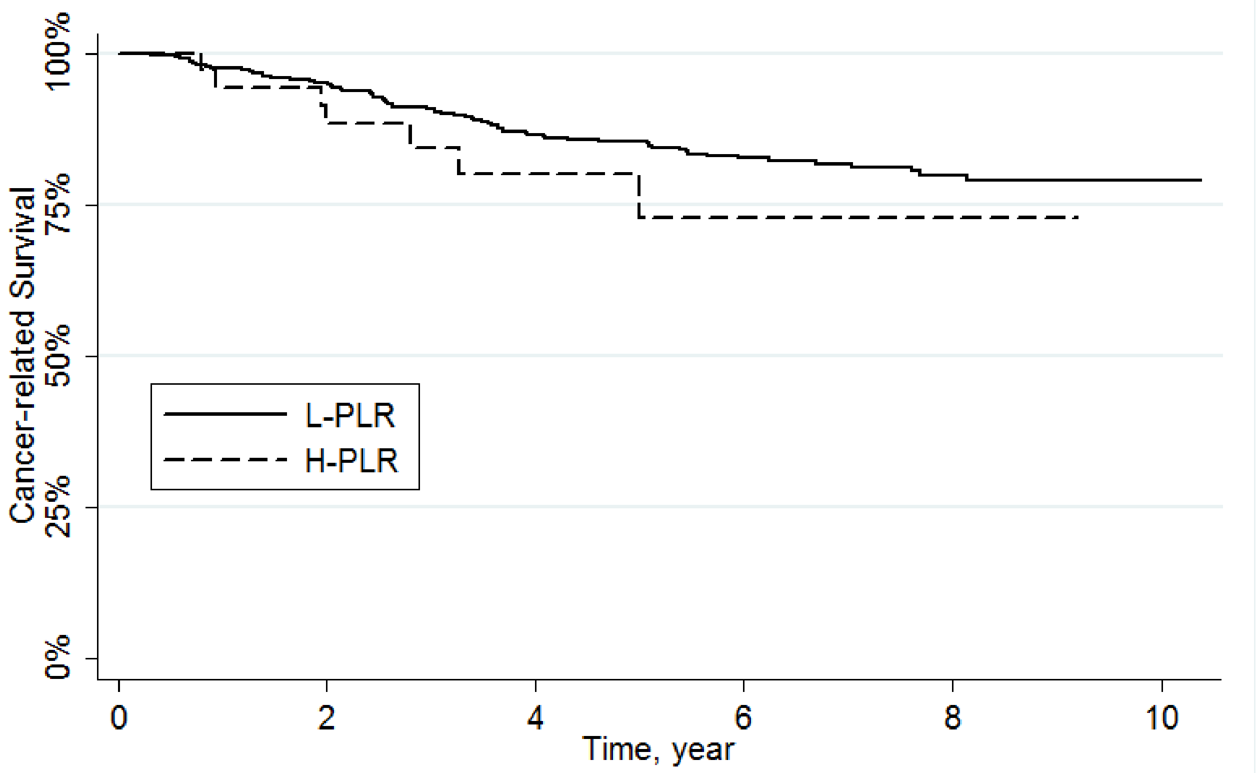
Cancer-related Survival
100%
75%
50%
25%
0%
L-PLR
H-PLR
0
2
4
6
8
10
Time, year

Figure S2(b)

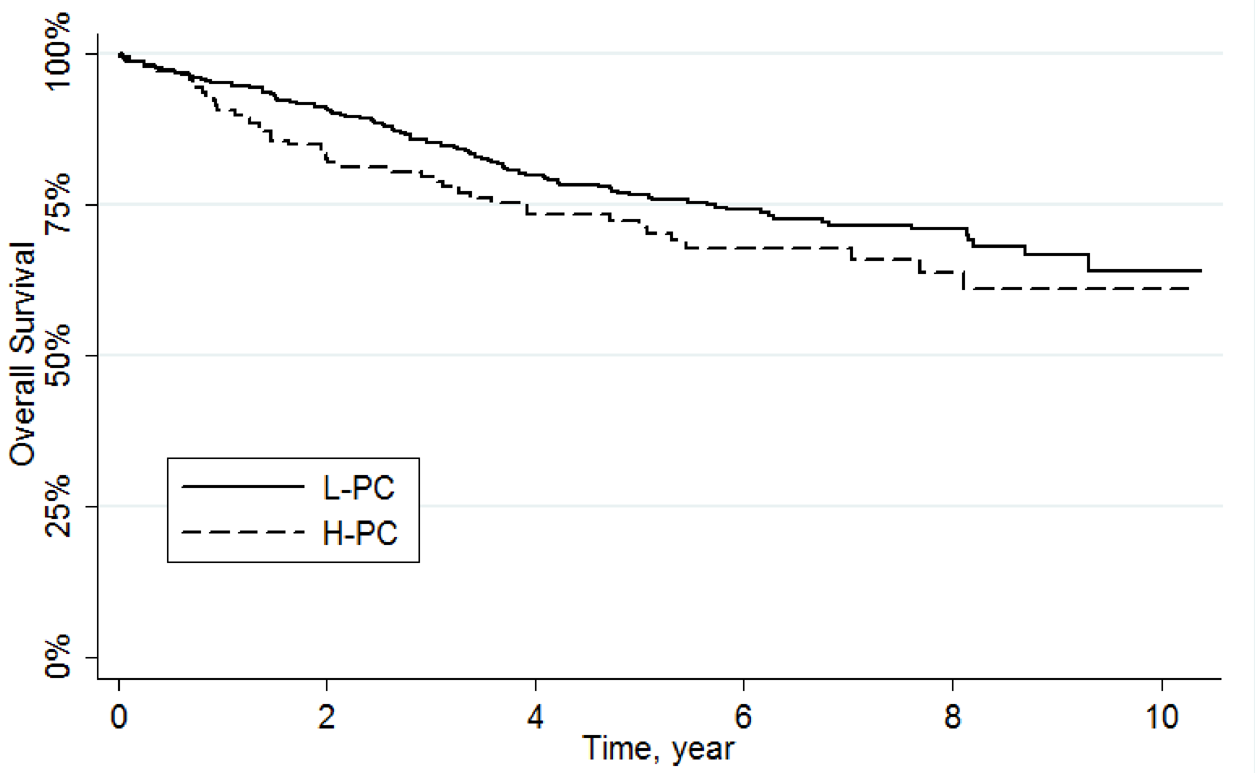

Figure S3(a)

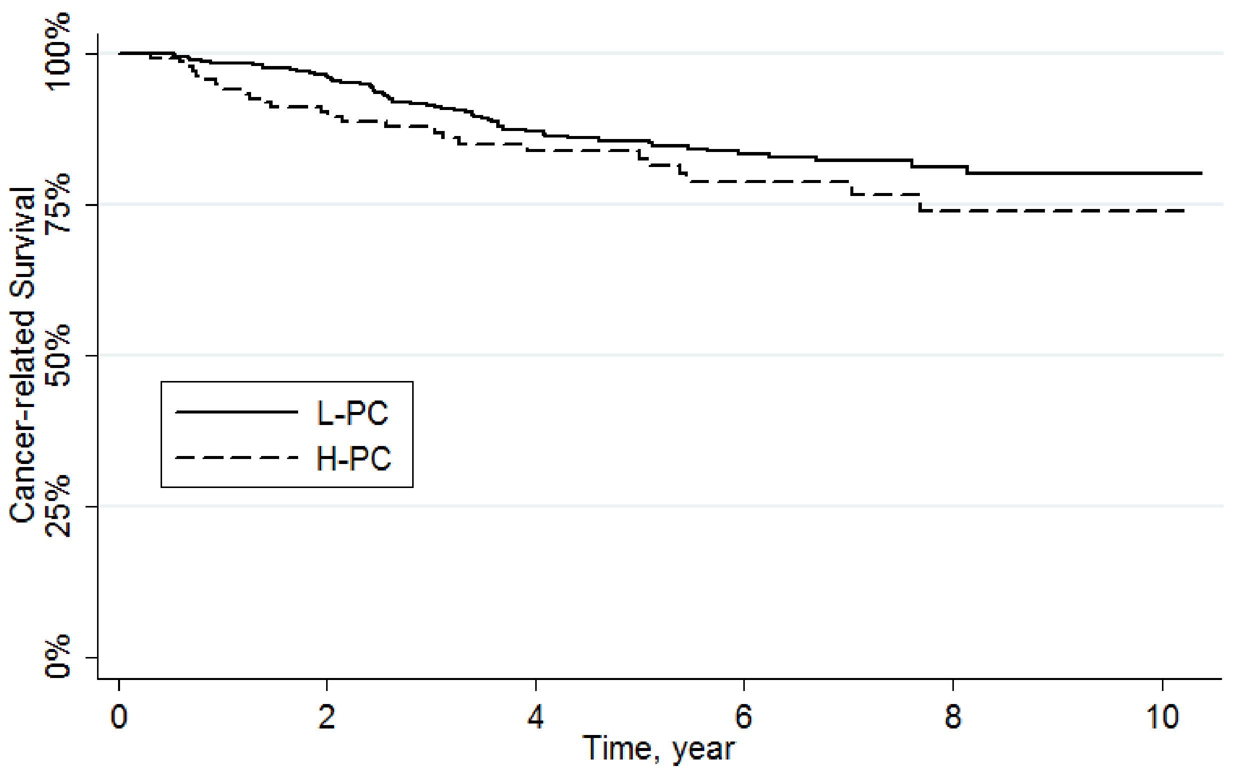

Cancer-related Survival
0%
25%
50%
75%
100%
L-PC
H-PC
0
2
4
6
8
10
Time, year

Figure S3(b)

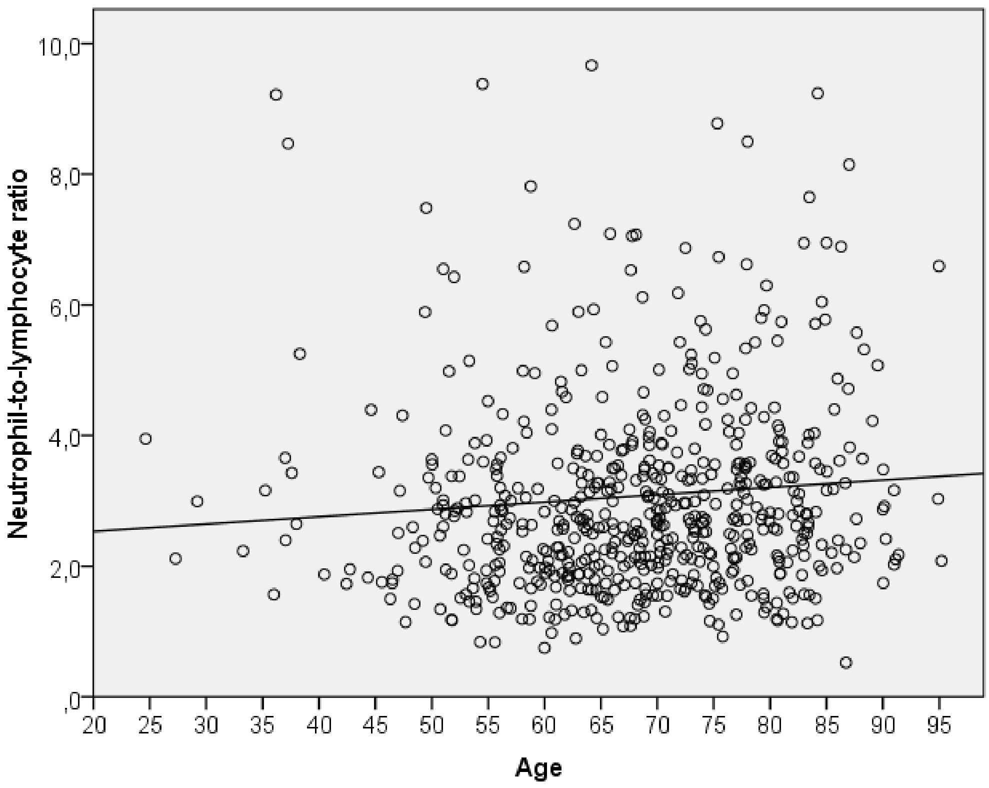

10,0
8,0
6,0
4,0
2,0
,0
Neutrophil-to-lymphocyte ratio
20
25
30
35
40
45
50
55
60
65
70
75
80
85
90
95
Age

Figure S4(a)

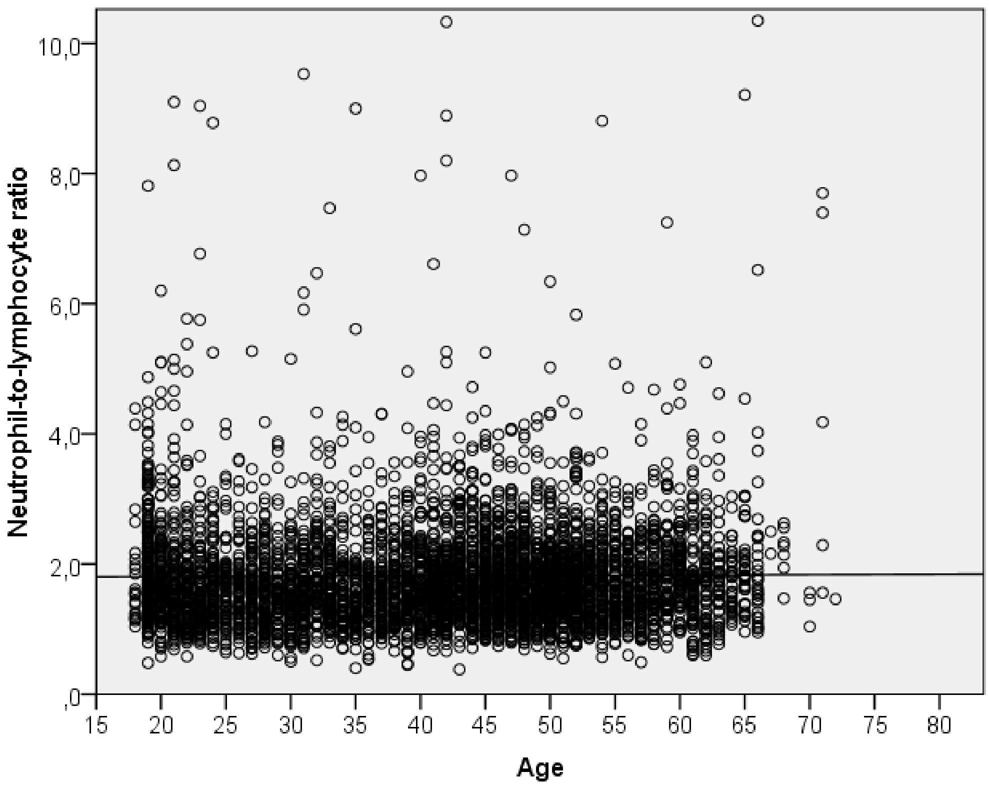

Neutrophil-to-lymphocyte ratio
10,0
8,0
6,0
4,0
2,0
,0
15 20 25 30 35 40 45 50 55 60 65 70 75 80
Age

Figure S4(a)

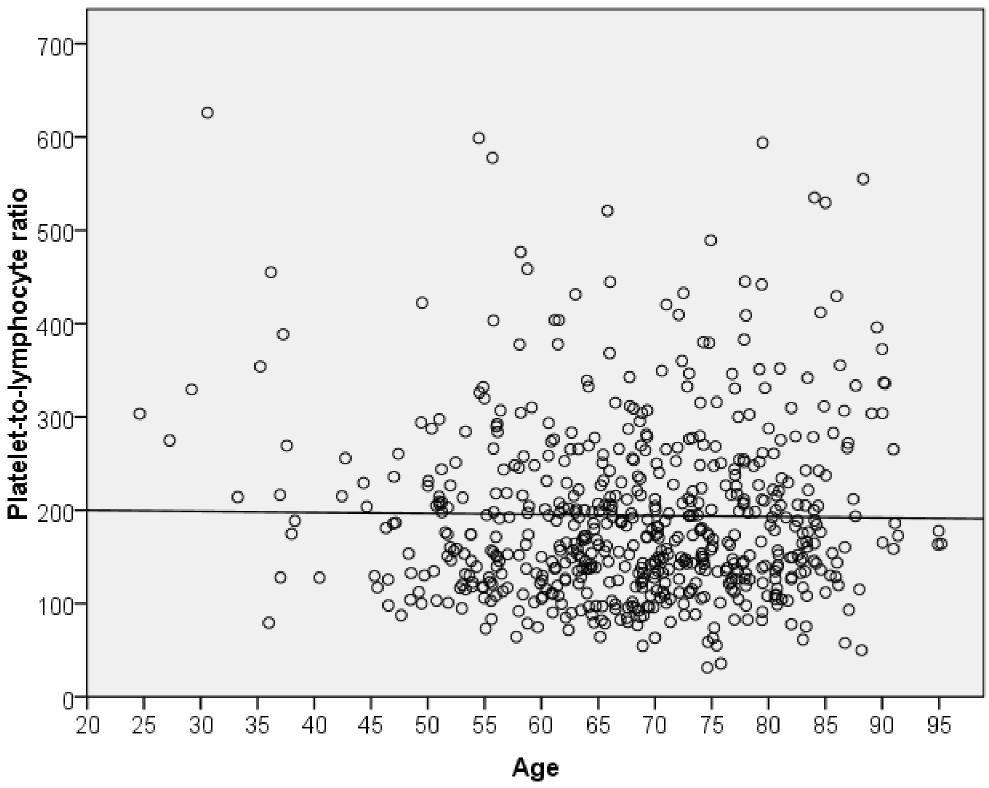
Platelet-to-lymphocyte ratio
700
600
500
400
300
200
100
0
20
25
30
35
40
45
50
55
60
65
70
75
80
85
90
95
Age

Figure S5(a)

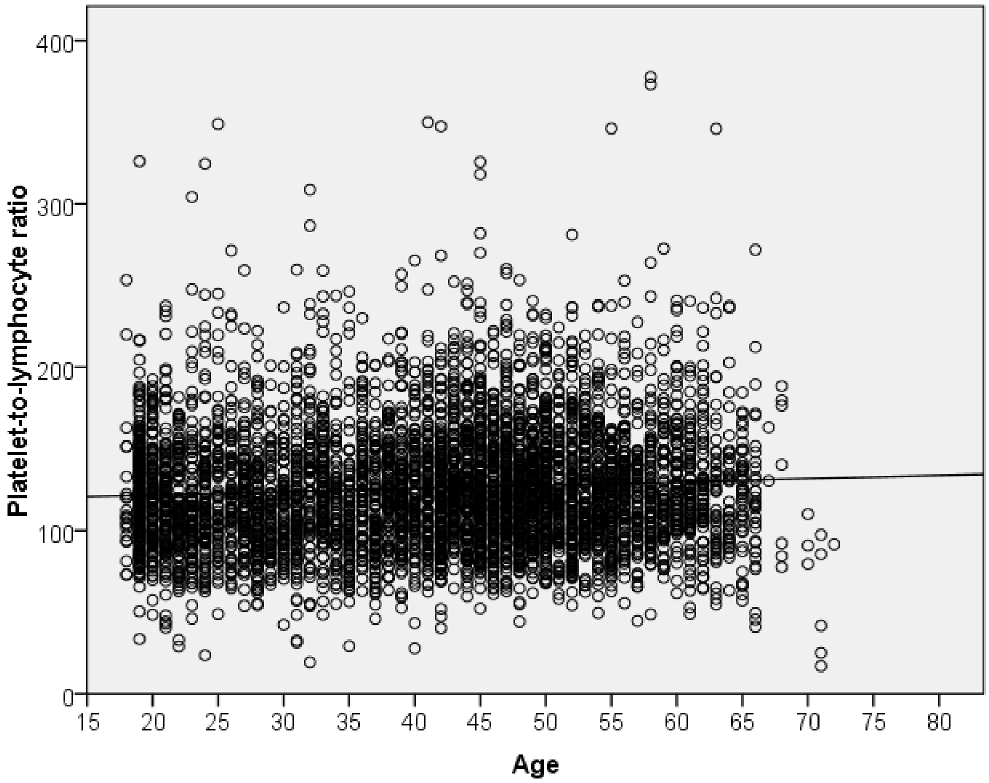

Platelet-to-lymphocyte ratio
400
300
200
100
0
15
20
25
30
35
40
45
50
55
60
65
70
75
80
Age

Figure S5(b)

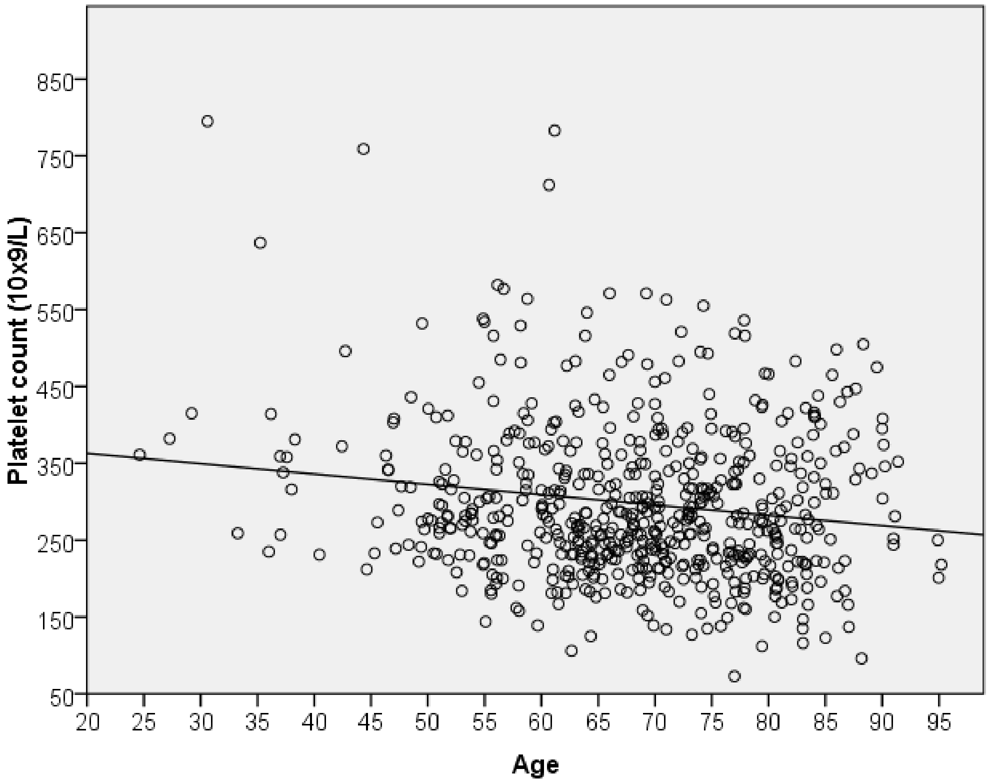
Platelet count (10x9/L)
850
750
650
550
450
350
250
150
50
20 25 30 35 40 45 50 55 60 65 70 75 80 85 90 95
Age

Figure S6(a)

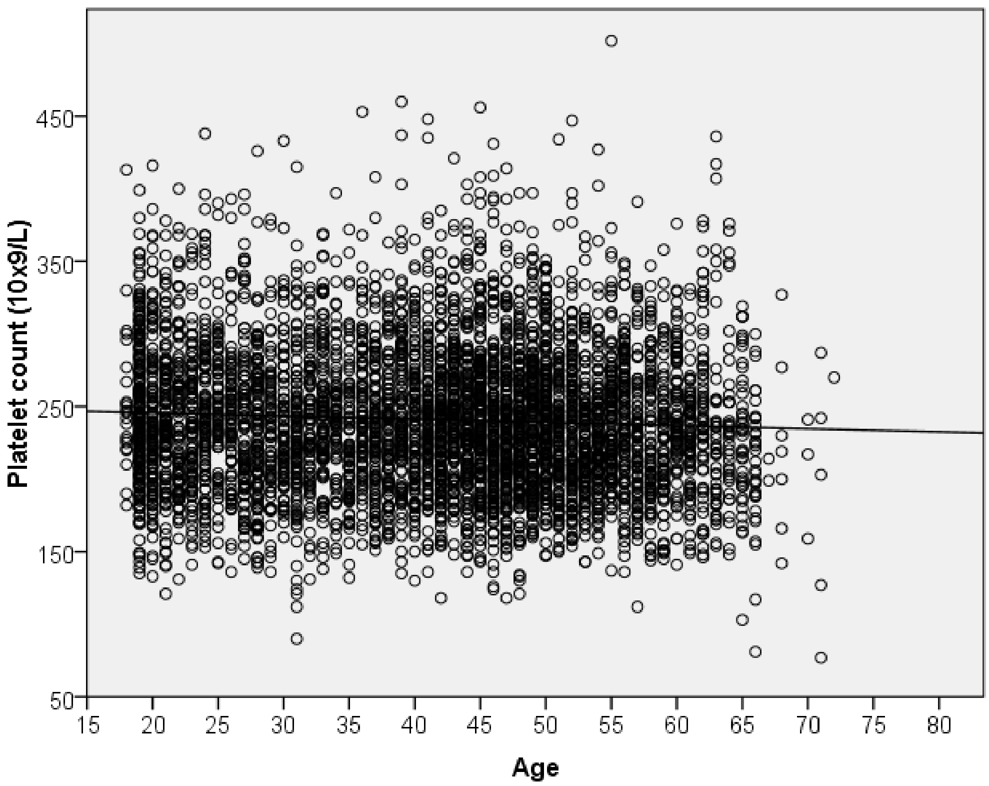
Platelet count (10x9/L)
450
350
250
150
50
15
20
25
30
35
40
45
50
55
60
65
70
75
80
Age

Figure S6(b)
